# Potential geographical distribution of *Garcinia paucinervis* Chun et How in China under future climate change scenarios based on the MaxEnt Model

**DOI:** 10.1371/journal.pone.0330483

**Published:** 2025-09-29

**Authors:** Hongqun Li, Lei Cheng, Junfang Song, Xieping Sun

**Affiliations:** 1 College of Modern Agriculture and Bioengineering, Yangtze Normal University, Chongqing, China; 2 Yan’an Huanglongshan Forestry Bureau, Yan’an, China; National Cheng Kung University, TAIWAN

## Abstract

*Garcinia paucinervis* Chun et How is a tree species with important ecological, medicinal, and ornamental value. Studying the impact of climate change on the potential distribution of this species offers important information for resource conservation, population restoration, and sustainability. In this study, the MaxEnt model was used to simulate potential distributions under climate change conditions. Results showed that the precipitation of the driest quarter (Bio-17) ranging from 33.3 to 133.3 mm, the precipitation of the warmest quarter (Bio-18) from 667.67 to 1000 mm, the annual mean temperature (Bio-01) from 18.0 to 24.0 °C, and the annual precipitation (Bio-12) from 1250.0 to 1760.0 mm were four dominant factors affecting the distribution of *G. paucinervis*. Its suitable habitat in China is the narrowest, and it is located in most regions of Guangxi and Guangdong, the southern region of Guizhou, and the southeastern part of Yunnan Province. In the 2050s and 2070s, the geographical distribution gradually decreased compared to current scenarios. Specifically, most of Guangxi and Guangdong, the southern region of Guizhou, the eastern part of Yunnan adjacent to Guangxi, the southeast region of Sichuan, and the northern region of Hainan were identified as stable suitable habitats for *G. paucinervis*. Meanwhile, the expanding areas were located only in the western and southern regions of Yunnan, and the contracting areas were in the junction of Guangdong, Fujian, and Jiangxi; among Guizhou, Chongqing, and Hunan; among Anhui, Henan, and Hubei; the southeastern region of Sichuan; the western region of Hubei; and the adjacent area between Chongqing and Sichuan. By the 2070s, the contracting habitats will additionally include the central Guizhou region, the northern regions of Guangdong and Guangxi, the eastern region of Guangdong adjacent to Fujian, and the southern Jiangxi Provinces. Thus, this study highlights the vulnerability of the species and its response to future climate change and provides insights for assessing habitat suitability for conservation management.

## Introduction

The rare and endangered plant *Garcinia paucinervis* Chun et How, belonging to the genus *Garcinia* in the family Clusiaceae, is a precious tree species unique to the karst mountains. It is mainly distributed in southwest Guangxi and southeast Yunnan Provinces and has economic, ecological, ornamental, and medicinal value [1,2]. For example, its branches and leaves are glossy, generally green with bright red young leaves, and it has a tall and beautiful tree shape, which makes it an excellent tree species for ornamental and greening purposes in gardens [2]. Concurrently, its leaves, branches, bark, and fruits can be used as medicinal materials to clear heat, detoxify, and reduce swelling; therefore, it is commonly used to treat sores, burns, and other conditions [3,4]. *G. paucinervis* is sturdy, corrosion-resistant, highly water-resistant, and not susceptible to insect infestation; therefore, some ancient buildings built with these materials are not easily eaten by insects and last for hundreds of years [5]. Owing to these excellent characteristics, it has become an important material in machinery, military, shipbuilding, construction, and high-end furniture since ancient times. Therefore, the local government has made it one of the characteristic resource plants with great development value in the karst mountainous regions [4,5]. In addition, the root system of *G. paucinervis* is relatively well developed and has good soil-water conservation effects, making it an important afforestation tree species in the karst mountainous areas [6]. In the past, because of the radiating golden wire-like rays in its cross-section, dense and orderly structure, and beautiful and straight texture of its trunk, *G. paucinervis* was previously subjected to excessive logging [7,8]. To date, because of the narrow distribution area, slow growth, excessive logging, and poor natural regeneration of *G. paucinervis*, and because its seeds do not germinate easily, it has gradually fallen into an endangered state [4,9]. Consequently, it is listed as a second-class key protected plant of China in the “List of Rare and Endangered Protected Plants in China” and classified as a vulnerable species in the “Red Book of Chinese Plants” [7,10]. In recent years, the deterioration of karst landforms has constrained the socioeconomic development of karst areas. *G. paucinervis* has already been considered an excellent tree species for vegetation restoration and urban greening in rocky mountain areas based on the above-mentioned characteristics [5,6]. However, little is known about the potentially suitable areas for *G. paucinervis*; therefore, farmers may grow it extensively in unsuitable areas, resulting in a series of problems without scientific guidance for cultivation areas. Therefore, to protect resources, restore natural populations, and promote the introduction and cultivation of this species, it is necessary to conduct research on its potential distribution and dynamic changes under climate change conditions.

Fortunately, species distribution models (SDMs) are reliable tools that are widely utilized by domestic and foreign scientists in many fields, such as endangered species conservation, protected area planning, and invasive species control, to assess species-surrounding relationships and predict the potential distribution area for a species [11,12]. Specifically, based on specific mathematical algorithms, these SDMs can calculate the ecological niche of a species and project it into a given geographical area to reflect the species’ preference for a certain habitat in the form of a probability distribution [13,14]. Among these SDMs, the MaxEnt model (maximum entropy, MaxEnt) was used to simulate the potential distribution of species based on maximum entropy principles, even with relatively small amounts of presence-only data and has already been demonstrated to outperform other models in multiple aspects, such as low distortion and good stability [15,16], and has recently been considered one of the best prediction models [16,17]. Importantly, the several merits of the MaxEnt model are as follows: (1) It only requires presence-only occurrence data coupled with environmental variables responsible for the potential distribution of species; (2) It effectively handles highly collinear environment variables, so collinearities have little negative effects on model performances [18,19]; (3) It requires only low computer specifications and has a user-friendly interface; (4) It has been confirmed to offer good prediction accuracy, even in the situation of incomplete data and/or small sample sizes [11,15]; (5) It can assess the weight of each environmental variable automatically. Meanwhile, according to the Fifth Assessment Report (AR5) of the Intergovernmental Panel on Climate Change (IPCC), global climate warming will continue, and the average temperature on the planet is projected to rise by 0.3–4.5°C through the end of the 21st century [20]. Consequently, climate warming may alter the potential distribution patterns of species, drive biodiversity loss, cause loss of germplasm resources, and even accelerate the extinction of species [21,22]. Therefore, studying the impact of climate warming on plant distribution is beneficial for agricultural production and biodiversity protection and can promote the sustainability of ecosystems.

Studying the response of species distribution patterns to climate change has important theoretical and practical value for resource protection and the development and utilization of this species. Therefore, to avoid investment risks from blindly expanding the potential cultivation zones, it is urgent to assess the potential spatial expansion of regions under climate change conditions. The aims were: (1) to determine the potential growing areas of this species under the current conditions and identify the most dominant variables responsible for the potential spatial distributions; and (2) to delineate the trends of the species’ growing regions with climate change, which will be helpful to governments at all levels to carry out regional planning of this species in China.

## Materials and methods

### Species distribution samples

The geographical distribution data from species specimen of *G. paucinervis* in the entire land area of China mainly came from two free databases: the Teaching Specimen Resource Sharing Platform (http://mnh.scu.edu.cn/), and the Chinese Virtual Herbarium(http://www.cvh.org.cn/).. Moreover, other distribution data for this species were mainly acquired from published scientific articles [2,6–8,23], which only provided small place names where it had been recorded. If so, the geographic coordinates(i.e., latitude and longitude) of the distribution points at the town or village level were accurately determined according to the Baidu Pick Coordinate System (http://api.map.baidu.com/lbsapi/getpoint/index.html) or the Gaode Pick Coordinate System (https://lbs.amap.com/tools/picker). To avoid errors from obvious misidentifications and high spatial autocorrelation from adjacent locations [24], some data that could not be accurately obtained from the geographical coordinates and duplicate points were deleted. To eliminate the overfitting error caused by clustering effects, only the distribution point closest to the center of each grid was ultimately selected from a grid with a 30-arc-second spatial resolution generated in the Data Management Tools-Create Fishnet of ArcGIS 10.2 [16,25]. Finally, a total of 74 distribution point were filtered from 141 data points for MaxEnt model construction. For compatibility with the software package of the MaxEnt model, the coordinates of each distribution point in the database were stored in CSV format according to the species name, longitude, and latitude.

### Environmental data

The distribution pattern of plants is mainly influenced by abiotic factors such as precipitation, temperature, soil, altitude, aspect, and slope [5,15]. Scientific evidence shows that climatic factors, such as precipitation and temperature, are the most important environmental factors affecting the distribution of species on a regional and global scale, and climate change has a significant impact on biodiversity and species distribution range [25–27]. These bioclimatic variables, considered biologically more meaningful than annual or monthly averages of climatic variables [28,29], are calculated from the minimum, maximum, and average values of monthly, quarterly, and annual ambient temperatures, as well as precipitation, reflecting a combination of annual changes, seasonal characteristics, and extreme environmental conditions [28,29]. These future bioclimatic variables were obtained from the BCC-CSM2-MR of the sharing economy path of the 6th phase of the International Coupled Model Intercomparison Project (CMIP6) in the WorldClim database (http://worldclim.org). To understand the dynamic changes, these bioclimatic variables were selected for the period 1970–2000 and the future periods of the 2050s (average for 2041–2060) and the 2070s (average for 2061–2080). Shared Socio-economic Pathways (SSPs) are a development scenario framework established by the Intergovernmental Panel on Climate Change (IPCC) of the United Nations, which can better reflect the relationship between economic and social development and climate change and gradually gain widespread application [17,30]. Compared to the Representative Concentration Pathways (RCPs) used in international research on the impact of climate change, the SSPs not only considers carbon emissions but also economic and social factors such as population, technology, education, and ecology. Herein, we first downloaded 19 bioclimatic variables (version 2.1) with a 30-arc-second (ca.1km^2^ at ground level) spatial resolution from the global Worldclim website(http://www.worldclim.org) under current (in the period–1970–2000) condition, which is equal to the average values from 1970 to 2000 [28,29]. Secondly, among global climate models (GCMs), BCC-CSM2-MR (Beijing Climate Center Climate System Model) released by China National Climate Center in CMIP6 has been proven to be more suitable for China’s climate change characteristics [16,17], so in the study, SSP1–2.6 (the low greenhouse gas emission scenario), SSP2–4.5 and SSP3–7.0 (the moderate greenhouse gas emission scenario), and SSP5–8.5 (the high greenhouse gas emission scenario) in two periods, 2050s (average for 2041–2060) and 2070s (average for 2061–2080) were selected because they can more scientifically describe future climate change [17,31]. Third, to obtain three topographic variables (elevation, aspect, and slope), we downloaded the Digital Elevation Model (DEM) data from the WorldClim website (http://www.worldclim.org) and produced slope and aspect data based on the DEM in the Spatial Analyst Tools of ArcGIS 10.2. Finally, based on the administrative boundary map of China from the national platform for common geo-spatial information service (https://www.tianditu.gov.cn), drawing review number: GS (2024) 0650, 22 environmental variables were extracted by the administrative boundary map of China from the above-mentioned global raster data and resampled with a 30-arc-second spatial resolution to ensure that all research layers have the same geographic boundary and cell size.

To reduce these negative effects on model building and to construct a model that has better performance with fewer variables, Spearman’s correlation analysis and percent contribution values were selected to screen the variables with considering the ecological characteristics of the species [12,17,32]. First, the MaxEnt model was selected to calculate the percent contribution of all environmental variables three times, and each time the variables whose percent contribution rate was equal to zero were removed. Second, the values of the 141 distribution points were extracted from the remaining corresponding layers in the Extraction-Extract Multi-Values to Points in ArcGIS 10.2. Thereafter, Spearman’s coefficients between two variables with a percent contribution greater than 0, corresponding to 141 distribution points of *G. paucinervis* were analyzed using SPSS(version 27.0). According to Spearman’s correlation coefficient (/r/ ≥ 0.8) in SPSS 27.0 and taking into consideration their relative predictive power (i.e., the percent contribution of all environmental variables), one variable with a higher percent contribution from each set of highly cross-correlated variables (/r/ ≥ 0.8) was kept in further analyses while the other variable was removed (Table 1). Otherwise, two environmental factors (/r/ < 0.8) remained (Table 1). Finally, a total of 10 remaining variables (Table 2) were screened and then utilized to establish the prediction model of *G. paucinervis*.

**Table 1:** The diagram of correlation between bioclimatic variables used in this study.

Variables	Bio-01	Bio-17	Bio-18	elev	Aspect	Bio-02	Bio-03	Bio-04	Bio-12	Bio-15
Bio-01	1.0									
Bio-17	−0.015	1.0								
Bio-18	0.132	0.246	1.0							
elevation	−0.464	−0.100	0.390	1.0						
Aspect	−0.057	0.223	0.083	0.298	1.0					
Bio-02	0.322	−0.215	−0.110	0.049	0.115	1.0				
Bio-03	0.683	−0.517	0.280	0.103	−0.052	0.515	1.0			
Bio-04	−0.413	0.761	−0.051	0.196	0.346	−0.015	−0.667	1.0		
Bio-12	−0.010	0.684	0.758	0.217	0.182	−0.158	−0.140	0.359	1.0	
Bio-15	0.295	−0.464	0.571	0.529	0.101	0.306	0.756	−0.486	0.072	1.0

Note: If two variables had >±0.8, only one of them with a higher percent contribution was selected in the model.

**Table 2:** The environmental factors used in this study, their contribution, and permutation importance under current conditions.

Code	Description	Percent contribution(%)	Permutation importance(%)	Code	Description	Percent contribution(%)	Permutation importance(%)
Bio-17	Precipitation of the driest quarter	**39.8**	**20.0**	Bio-02	Mean diurnal range	3.0	0.7
Bio-18	Precipitation of the warmest quarter	**29.3**	**55.7**	Bio-12	Annual precipitation	2.8	**7.4**
Bio-01	Annual mean temperature	**10.2**	**11.0**	Bio-03	Isothermality	2.6	0.4
Bio-15	Precipitation seasonality	5.6	1.2	Bio-04	Temperature seasonality	2.2	1.5
elev	elevation	3.4	1.8	Aspect	Slope direction	1.0	0.3

Note: The highlighted variables selected based on their contributions and permutation importance, were the four main influencing factors.

### Modelling process

The MaxEnt model, which has demonstrated superior performance in prediction accuracy even with limited distribution data [16,33], has been applied to predict the potential distribution area for species in China [25,34]. The MaxEnt model (version 3.4.4) can be freely downloaded from the American Museum of Natural History (https://biodiversityinformatics.amnh.org/open_source/maxent/). When modeling construction, the latitude and longitude information of 141 remaining species distribution points and 10 environmental variables screened were directly imported into the “sample” and “environmental layers” boxes of this model, respectively. The MaxEnt parameters were set as follows: 75% of all the distribution data were randomly selected for model training, and the remaining 25% for model validation [25,35]. Meanwhile, “Create response curves” and “Do jackknife to measure variable importance”were checked in the model interface to analyze the relationship between variables and presence probability, and estimate the importance of each environmental variable. The output format and type were set to “logistic” and “asc” in the interface of this model. To ensure the stability of prediction results, the “replicates” were set to 10 by cross-validation for model validation, so the final output result was the average of 10 prediction results in logistic format and asc types [25,34]. The other settings were the same as described in some scientifically published articles [12]. Subsequently, this output result was converted into a raster format in Conversion Tools-ASCⅡto Raster in ArcGIS 10.2, and the cell value of the logistic output result ranged from 0, representing the lowest habitat quality for this species, to 1, representing the highest habitat quality. Finally, the logistic output produced a species distribution map that provides the predicted probabilities of presence ranging from 0 to 1. In addition, the Maximum Youden Index (maximum training sensitivity plus specificity logistic threshold, an available output of MaxEnt) is usually taken as the cutoff point (threshold value), which provides advantages over other threshold values [17,36]. Finally, based on the above cut-off points (greater (suitable) or less than (unsuitable)), the continuous habitat areas were transformed into suitable and unsuitable habitats. Thereafter, we assigned them new pixel values; for example, the suitable habitat is assigned as 1, and the unsuitable habitat is assigned as 2 under current conditions, while under future climate change, the suitable habitat is assigned as 3, and the unsuitable habitat is assigned as 4. Then, two sets of raster data for different periods were multiplied in the Map Algebra-Raster Calculator in ArcGIS 10.2, and overlapping grids of different quality habitats were obtained. Specifically, a pixel value of three is not suitable for the two periods; four indicates a suitable habitat for expansion, six indicates a suitable habitat for contraction in future climate change, and eight indicates a suitable habitat for the two periods. Finally, all habitat area types were calculated after different projection conversion (https://blog.csdn.net/weixin_42160645/).

### Model performance and influencing factors

The area under the receiver operating characteristic (ROC) curve (AUC) is widely regarded as an excellent index to evaluate the performance of this model in ecological studies [15,37]. In general, the larger the AUC, the better the model performance. It is now generally believed that AUC = 0.5, implying that the prediction performance was not better than that of the random model or the lowest predictive ability, whereas AUC = 1.0, indicating the best performance or highest predictive ability. In our study, the AUC ranging from 0.5 (random accuracy) to 1.0 (perfect discrimination) was selected to evaluate the prediction accuracy of the model. Specifically, the evaluation criteria of the AUC value may be classified as 0.9–1(excellent), 0.8–0.9 (good), or 0.7–0.8(ordinary), respectively [25,36]. Based on the predictor contributions, permutation importance, and regularized training gain, the relative influence of individual predictors can be evaluated for species habitat suitability [25,37,38]. Furthermore, a response curve generated automatically from the modelling process was applied to analyze the relationships between environmental variables and habitat suitability for *G. paucinervis* [12,20].

## Result

### Evaluation of MaxEnt models

In this study (Table 3), inputting 80 distribution points of *G. paucinervis* and the screened 10 environmental variables into MaxEnt model and repeating the operation 10 times by cross-validation each time, the mean AUC values under current conditions were near 1.0 (0.991 ± 0.000 for the model building and 0.985 ± 0.004 for testing respectively). The mean AUC values of the model building under the future climate scenarios were 0.989 ± 0.000 ~ 0.991 ± 0.000 and those of the test data were 0.984 ± 0.003 ~ 0.987 ± 0.003. These results indicate that the prediction performance of the MaxEnt model was excellent, and all these models obtained satisfactory results in forecasting the potential habitat for this species.

**Table 3:** The AUC of model building and testing, and Maximum Youden Index generated in 10 replicates under climate change.

GCMs	Periods	AUC of model building	AUC of model testing	Mean Maximum Youden Index
	1970-2000	0.991 ± 0.000	0.985 ± 0.004	0.0441
SSP1–2.6	2050s^a^	0.990 ± 0.000	0.986 ± 0.003	0.0788
SSP2–4.5		0.998 ± 0.000	0.985 ± 0.003	0.1256
SSP3–7.0		0.991 ± 0.000	0.984 ± 0.004	0.0751
SSP5–8.5		0.991 ± 0.000	0.987 ± 0.003	0.0559
SSP1–2.6	2070s^b^	0.989 ± 0.000	0.984 ± 0.003	0.0742
SSP2–4.5		0.989 ± 0.000	0.981 ± 0.004	0.1315
SSP3–7.0		0.989 ± 0.000	0.986 ± 0.003	0.0943
SSP5–8.5		0.989 ± 0.000	0.981 ± 0.006	0.0451

^a^2050s = average for 2041–2060; ^b^2070s = average for 2061–2080.

### Suitable habitat under current climate condition

**Fig 1:**
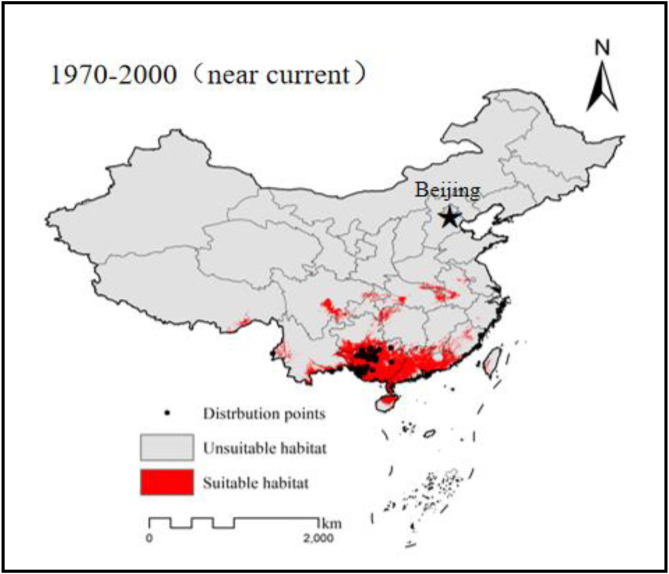
Suitable habitat simulated by MaxEnt model under current condition.

After completing the simulation of the potential distribution of *G. paucinervis*, we converted the predicted result from the output file type (ASC) to a raster format(TIFF) and reclassified it into suitable and unsuitable habitats based on the Maximum Youden Index. Based on the statistical calculations after coordinate projection (Fig 1) (Asia_North_Albers_Equal_Area_Conic), the results computed using ArcGIS 10.2 showed that the total suitable area of *G. paucinervis* in China was 54.7084 × 10^4^ km^2^, accounting for 5.77% of the total land area of China, indicating that the distribution area of the suitable habitat was relatively narrow and was mainly distributed in the southern part of China. Specifically, suitable habitats were located in most regions of Guangxi and Guangdong, the northern region of Hainan, the southeastern region of Guizhou adjacent to Guangxi, the southeastern and western regions of Yunnan, the southeastern narrow area of Sichuan, the northwestern region of Hunan, the northeastern region of Chongqing, the southwestern region of Hubei, and the junction area among Hubei, Henan, and Anhui (Fig 1).

### Importance of current environmental variables and their threshold

**Fig 2:**
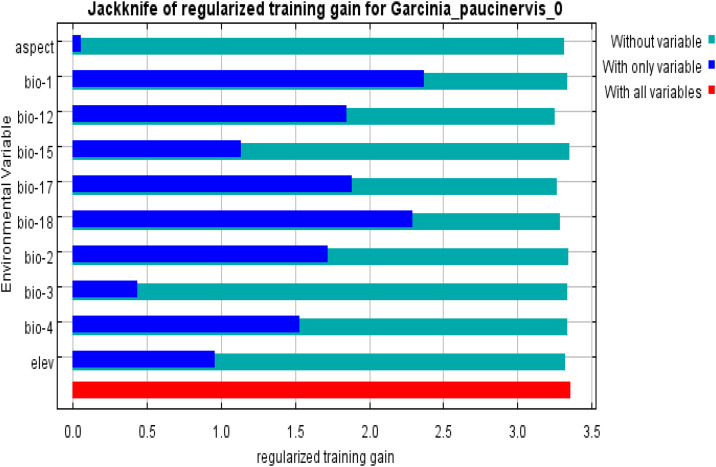
Jackknife test for assessing the relative importance of different environmental variables to the geospatial distribution of *G. paucinervis* under the current condition.

**Fig 3:**
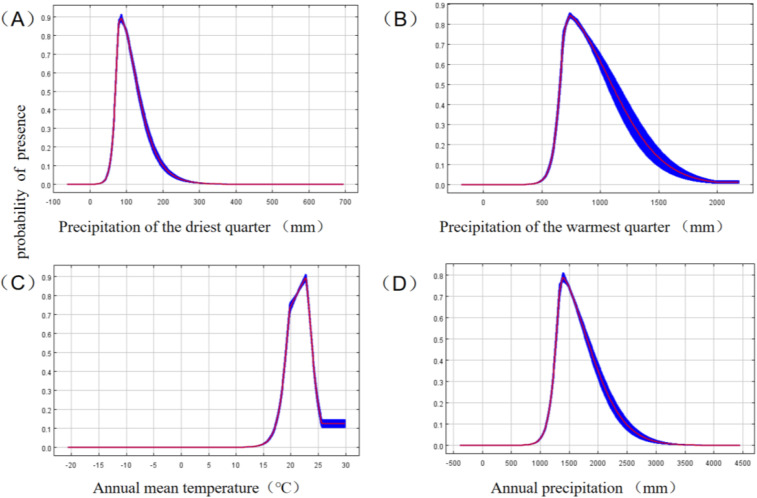
Response curves of the occurrence probability of *G. paucinervis* to the precipitation of the driest quarter (Bio-17), the precipitation of the warmest quarter (Bio-18), the annual mean temperature (Bio-01) and the annual precipitation (Bio-12).

According to the percent contribution, among a total of the remaining 10 environmental variables selected, precipitation of the driest quarter (Bio-17), precipitation of the warmest quarter (Bio-18) , and annual mean temperature (Bio-01) were the strongest predictors in the MaxEnt model relative to the contributions of the other environmental variables (Table 2). Precipitation of the driest quarter (Bio-17) made the largest contribution (39.8%), indicating that this variable significantly affected the distribution of *G. paucinervis* under the current conditions, followed by precipitation of the warmest quarter (Bio-18) and annual mean temperature (Bio-01) at 29.3% and 10.2%, respectively. The cumulative contribution of the top three key factors amounted to 79.3%. Based on the permutation importance (Table 2), precipitation in the warmest quarter (Bio-18) with 55.7% had the highest score (Table 2), followed by precipitation in the driest quarter (Bio-17), annual mean temperature (Bio-01), and annual precipitation (Bio-12) with 20.0%,11.0%, and 7.4%, respectively. The cumulative permutation importance of the top four key parameters was 94.1%. In addition, based on the built-in jackknife test of the software (Fig 2), the precipitation of the driest quarter (Bio-17), precipitation of the warmest quarter (Bio-18), annual mean temperature(Bio-01), and annual precipitation (Bio-12) had the highest regularized training gain for the geospatial distribution of *G. paucinervis*. Through a comprehensive analysis, the four key environmental variables selected were precipitation of the driest quarter (Bio-17), precipitation of the warmest quarter(Bio-18), annual mean temperature(Bio-01), and annual precipitation (Bio-12), which were mainly responsible for the distribution of *G. paucinerviss.* Using the response curve (Fig 3), we determined the thresholds (probability of presence >0.5) for the four key environmental variables. As shown in Fig 3, all 4 key factors exhibit a circular dome pattern; for example, the precipitation of the driest quarter (Bio-17) ranges from 33.3 to 133.3 mm, with a peak value of 71.5 mm; the precipitation of the warmest quarter (Bio-18) ranges from 667.67 to 1000 mm, with a peak value of 750 mm; the annual mean temperature (Bio-01) ranges from 18.0 to 24.0 °C, with a peak value of 22.5 °C and the annual precipitation (Bio-12) ranges from 1250.0 to 1760.0 mm, with a peak value of 1400 mm.

### Changes in distribution of suitable habitats under climate change scenarios

**Fig 4:**
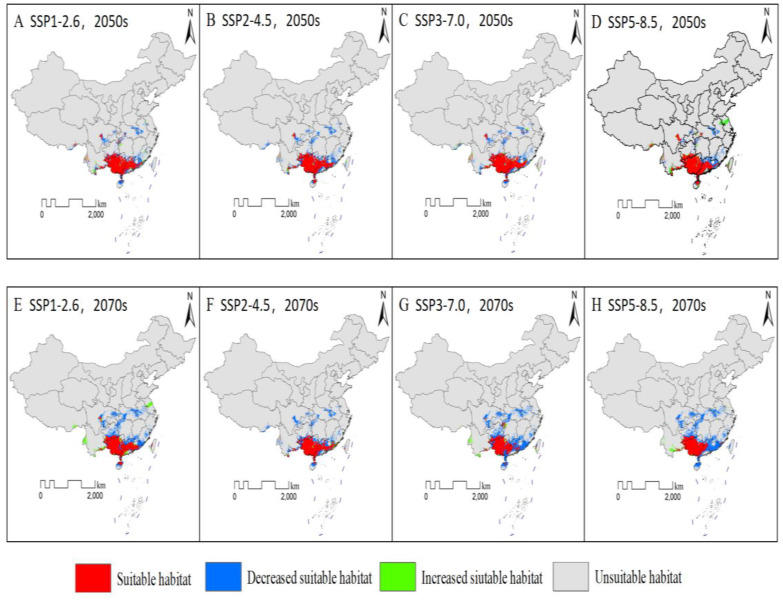
Changes in distribution of suitable habitats under climate change scenarios.

According to the Maximum Youden Index from various models, the continuous prediction results were classified into “suitable habitat” and “unsuitable habitat.” In the 2050s and the 2070s, the suitable geographic distribution pattern for *G. paucinervis* was basically consistent with that in the period–1970–2000; namely, it was still mainly distributed in southern China, but the distribution area will undergo some changes (Fig 3 and Table 4). Under SSP1–2.6, the area of the total suitable habitat was 43.74 × 10^4^ km^2^ (2050s) and 39.86 × 10^4^ km^2^ (2070s), which was decreased by 25.47(% (2050s) and 40.17.54% (2070s) compared with the current situation, respectively. Under SSP2–4.5, the total suitable habitat was 38.79 × 10^4^ km^2^ (2050s) and 36.31 × 10^4^ km^2^ (2070s), which decreased by 32.53% (2050s) and 37.03% (2070s), respectively, compared with the current situation. Under SSP3–7.0, the total suitable habitat was 43.52 × 10^4^ km^2^ (2050s) and 22.74 × 10^4^ km^2^ (2070s), which decreased by 25.47% (2050s) and 65.17% (2070s) compared with the current situation, respectively. Under SSP5–8.5, the total suitable habitat was 49.55 × 10^4^ km^2^ (2050s) and 22.04 × 10^4^ km^2^ (2070s), which was a decrease of 9.43% (2050s) and 59.71% (2070s), respectively, compared with the current situation. The results showed that the geographical distribution area gradually decreased under climate change because the reduced area was greater than the increased area in the study area (Table 4). Specifically, Fig 3A–3D shows a stable, expanding, and shrinking area of suitable habitat in the 2050s compared with the current situation. The results showed that most of Guangxi and Guangdong, the southern region of Guizhou, the eastern region of Yunnan adjacent to Guangxi, the southeastern region of Sichuan, and the northern region of Hainan were identified as stable suitable habitats for *G. paucinervis*. The expanded habitat had an area of 4.374 × 10^4^ km^2^(18786.1–84152.5), which was relatively concentrated only in the western and southern regions of Yunnan. The area of the shrinking habitat was 16.71 × 10^4^ km^2^ (135763–196760), which was located at the junction of Guangdong, Fujian, and Jiangxi; the junction area among Guizhou, Chongqing, and Hunan; the southeast region of Sichuan; the western region of Hubei; the adjacent area between Chongqing and Sichuan; and the junction area between Anhui, Henan, and Hubei. Fig 3E–3H shows the stable, expanding, and shrinking areas of suitable habitat in the 2070s compared with the current situation. The results showed that the distribution area of stable habitat was consistent with that in the 2050s (Fig 4). Compared with the 2050s, the expanded habitat will show an increasing trend, especially in the western and southern regions of Yunnan. While shrunken habitats except for the above-mentioned regions, will exist in central Guizhou, northern Guangdong, Guangxi adjacent to Hunan, eastern Guangdong adjacent to Fujian, and southern Jiangxi, which presents a larger reduction area. On the whole, the distribution area of *G. paucinervis* decreased with the climate change.

**Table 4:** Changes of the suitable habitat for *G. paucinerviss* by the period of 2041-2060 (2050s) and 2061-2080 (2070s).

Climate scenarios	Comparative periods	Decreased suitable habitat		Increased suitable habitat		Unchanged suitable habitat		Total habitat change	
		Area/ km^2^	Percent/ %	Area/ km^2^	Percent/ %	Area/ km^2^	Percent/ %	Area/ km^2^	Percent/ %
SSP1–2.6	Now-2050s	169101	30.91	29744.7	5.44	407727.7	74.53	139356.3	25.47(–)
	Now-2070s	291056	53.20	71294.8	13.03	327322.8	59.83	219761.2	40.17(–)
SSP2–4.5	Now-2050s	196766	35.97	18786.1	3.43	369104.1	67.47	177979.9	32.53(–)
	Now-2070s	221168	40.43	18597.8	3.40	344513.8	62.97	202570.2	37.03(–)
SSP3–7.0	Now-2050s	166740	30.48	27409.2	5.01	407753.2	74.53	139330.8	25.47(–)
	Now-2070s	393405	71.91	36853.6	6.74	289850	52.98	356551.4	65.17(–)
SSP5–8.5	Now-2050s	135763	24.82	84152.5	15.38	408418	74.65	51610.5	9.43(–)
	Now-2070s	189092.9	34.56	89798.6	16.41	353394.4	64.59	99294.3	18.15(–)

Note: the suitable areas under current situation is 547,084 km^2^; + /–: increase/decrease

## Discussion

The rare and endangered plant *G. paucinervis* unique to the karst mountains, is a precious tree species with high economic value because of its timber, ornamental, and medicinal uses [6,7]. In recent years, the deterioration of karst habitats has constrained the socioeconomic development of karst areas. For *G. paucinervis,* due to its well-developed root system and effective soil-water conservation*,* it has already been considered as an excellent tree species for vegetation restoration and urban greening in limestone mountainous areas [2,8]. To protect resources, restore the natural population, and promote the introduction and cultivation of this species, it is necessary for governments at all levels to explore the most suitable growth zones. Fortunately, with recent developments in applied ecology and statistical biology, many SDMs including mechanistic, regression, and niche models, have been widely adopted to identify the potential distribution of many species [11,12]. Among them, the mechanistic model has the disadvantage of relying on expert experience and is difficult to verify correctness, and the reliability of non-occurrence points in a regression model is often difficult to guarantee [15], so the application of these two models are restricted to a certain extent. Compared with mechanistic and regression models, the niche model is widely applied for accurately forecasting species distribution because of its avoiding the subjectivity of mechanistic models and the difficulty in obtaining non-occurrence data for regression models and [39,40]. However, among the niche models the domain model has been used to estimate the potential distribution of *Garcinia* based on nineteen bioclimatic variables at a spatial resolution of 2.5 arc-min [41]. Consequently, the obtained results are not detailed enough to guide relevant work due to its high resolution, and the research area is only limited to Guangxi and Yunnan Provinces. In this study, potential distributions were simulated using the MaxEnt model under climate change conditions based on only 80 precise coordinates of species occurrence and screened 10 environmental layers with a 30-arc-second spatial resolution. All of these results were excellent, with AUCs > 0.9 for model training and testing, which indicated that these results were excellent in forecasting the potential habitat for this species. Furthermore, based on the above-mentioned prediction outcomes (Fig 1), the suitable area was generally consistent with the actual distribution and literature reports of *G. paucinervis* [6,8,41], indicating that the predictions were exceptionally precise, and the above threshold value under the current conditions was reasonable and reliable. Compared with a previous report (in the southwest of Guangxi and southeast Yunnan Province) [6,8,41], most regions of Guangdong and Guangxi(except for its southwest region), the southern region of Guizhou, and the western and eastern region of Yunnan were identified as newly added suitable areas, indicating that *G. paucinervis* has not yet reached its full potential range (Fig 1), accounting for 5.77% of the total land area of China. This indicates that the distribution area of the suitable habitat was relatively narrow. As shown in Fig 1, under the current conditions, the ideal distribution area of *G. paucinervis* was mainly concentrated in most regions of Guangxi and Guangdong, the northern region of Hainan, the southeastern region of Guizhou adjacent to Guangxi, the southeastern and western regions of Yunnan etc. Therefore, we recommend enhanced conservation efforts in current prime habitats and explored potential cultivation in newly identified suitable regions under the current conditions.

The spatial distribution of a species is mostly affected by temperature, precipitation, terrain, and other factors [12,42]. Among them, climate is one of the most notable factors responsible for the spatial distribution of species on Earth [12,25,43]. In this study, based on the predictor contributions, permutation importance and regularized training gain, the four key environmental variables responsible for the distribution of *G. paucinervis* were the precipitation of the driest quarter (Bio-17) from 33.3 to 133.3 mm, the precipitation of the warmest quarter (Bio-18) from 667.67 to 1000 mm, the annual mean temperature (Bio-01) from 18.0 to 24.0 °C and the annual precipitation (Bio-12) from 1250.0 to 1760.0 mm, which indicated that temperature and precipitation jointly constrain the geographical distribution of *G. paucinervis* in China. This is in agreement with the results of a previous study in which seed germination was sensitive to temperature range and water permeability [8]. For example, the seeds could germinate at 25 °C, 32 °C, and room temperature, and the germination process and growth rate could be obviously accelerated at 32 °C, but the seeds did not germinate at 18 °C and 37 °C, which indicated that the seeds could germinate from 18 °C to 37 °C. Furthermore, *G. paucinervis* is distributed in areas with an annual average temperature range of 20−22°C, indicating that it prefers a warm and humid environment with abundant rainfall, as well as loose and moist neutral or slightly alkaline limestone soil [6]. This is consistent with our results (for example, the annual mean temperature (Bio-01) from 18.0 to 24.0 °C). The distribution area has abundant rainfall, with a minimum annual precipitation of 1201.1 mm (Chongzuo Jiangzhou District) and a maximum of 1887.6 mm (Hekou Nanxi Town), mostly concentrated from April to September [6]. According to a previous report [8], the ripening period of the fruit coincides with hot and rainy summers in karst mountain areas. Consequently, some seeds germinate in the same year under suitable conditions, whereas non-germinating seeds are buried in the soil as potential populations because of factors, such as animal trampling, rainwater, and litter decomposition, forming a soil seed bank to survive low temperatures and dry winter [44]. Furthermore, owing to abundant rainfall and strong leaching in the distribution area, loose soil with high nitrogen and phosphorus contents in the weathered surface soil layer of karst mountains is beneficial for the growth of this plant [6]. This proves that our research results are correct, namely, the annual precipitation (Bio-12) from 1250.0 to 1760.0 mm and the precipitation of the warmest quarter (Bio-18)(probably in summer) from 667.67 to 1000.0 mm. Usually, soil not only provides nutrients for plants but also maintains moisture. *G. paucinervis* is distributed in limestone mountains of karst landforms, such as peak clusters and valleys, and the surface soil layer formed after weathering is relatively shallow and has poor moisture retention [45,46]. However, the peak cluster depression and valley have a good water retention effect due to their low terrain, which can adapt to the precipitation of the driest quarter (Bio-17) from only 33.3 to 133.3 mm for *G. paucinervis*.

According to our findings, the suitable distribution area will gradually decrease by 9.43–32.53% in the 2050s, or 18.15–65.17% in the 2070s (Table 3, Fig 4), which indicates that global climate change will exacerbate the loss of resources for *G. paucinervis*. According to the past report [32], there was a projected expansion of suitable habitats compared to current scenarios, suggesting a positive regulatory effect of temperature changes induced by elevated carbon dioxide concentrations within a certain threshold. As shown in panel C of Fig 2, at annual mean temperature above 15.0°C, the probability of *G. paucinervis* occurrence increased sharply. At a temperature of 22.50 °C, the possibility of *G. paucinervis* occurrence peaked and started to decline(Fig 2C). Namely, within 22.50 °C, suitable habitats increases, while more than 22.50 °C, suitable habitats reduces with the increase of temperature in the 2050s and 2070s. Meanwhile, based on the variation of the annual precipitation (Fig 3), within 1400 mm, suitable habitats increases, while more than 1400 mm, suitable habitats reduces with the increase of the annual precipitation in the 2050s and 2070. These results indicated that within a certain threshold, with the increase of annual precipitation and temperature, the suitable habitat area for *G. paucinervis* increases, if more than a certain threshold, suitable habitats reduces. Additionally, under conditions of global warming, many low slopes of rocky mountains have developed into farmlands [6], posing more serious challenges to the survival of *G. paucinervis*. As shown in Fig 4 and Table 3, the expanded habitat was relatively concentrated only in the western and southern regions of Yunnan, while the shrunken habitats were at the junction of Guangdong, Fujian, and Jiangxi; among Guizhou, Chongqing, and Hunan; the southeastern region of Sichuan; the western region of Hubei; the adjacent area of Chongqing and Sichuan; and the junction area among Anhui, Henan, and Hubei. By the 2070s, the shrink habitats will increase in the central Guizhou region, northern Guangdong and Guangxi adjacent to Hunan, eastern Guangdong adjacent to Fujian, and southern Jiangxi. The future stable rate was 67.47%−74.53% in the 2050s and 52.98%−64.59% in the 2070s, and most of these areas were located in most regions of Guangxi and Guangdong, the northern region of Hainan, the southeastern region of Guizhou adjacent to Guangxi, the southeastern regions of Yunnan etc, which is closely consistent with the current predicted suitable areas by the MaxEnt model. Therefore, in the future, stable and expanded habitats, may be used for the protection, restoration, and development of *G. paucinervis* while for the unsuitable and shrunken habitats mentioned above, its development and utilization should be avoided to reduce the investment risk of blind expansion.

Previous studies have found that the possible reasons for the endangered status of *G. paucinervis* are as follow [6,8]: (1) the slow germination speed and growth of seedlings are not conducive to the rapid occupation of population space resources, and seed germination has a higher requirement for soil permeability, moisture and temperature range; (2) The vegetation of its community has been destroyed historically by human activities with higher economic value, so the seedlings died under strong light irradiation caused by excessive deforestation; (3) In their original habitat, there are very few big trees that bear fruit, so the seed resources are valuable [6]. Additionally, the fruit is easily eaten by monkeys and other animals, and has a short lifespan. In addition, although their seeds are large, they do not germinate easily, indicating that their seed resources are limited [6,10]. This plant has no sprouting phenomenon, and it is extremely difficult to propagate through cuttings [8]. It has been speculated that sexual reproduction(seeds) is the main mechanism for its propagation. Based on some of the reasons, resource conservation faces severe challenges. The results of our study indicate that with climate warming, suitable distribution areas will gradually decrease, implying that global climate change will exacerbate the loss of resources for *G. paucinervis.* Based on the suitability zone map and spatial transformation pattern from our study, most of Guangxi and Guangdong, the southern region of Guizhou, the western and southern regions of Yunnan adjacent to Guangxi, the southeastern region of Sichuan, the northern region of Hainan, and the east-west and southern regions of Yunnan were identified as suitable habitats for *G. paucinervis* and should be recommended as priority protection zones. In the 2050s and 2070s, the stable and expanded habitats may be used for the protection, restoration, and development of *G. paucinervis* while these unsuitable and shrink habitats should avoid its development and utilization to reduce the investment risk of blind expansion. Furthermore, a three-dimensional protection model combining ex situ and in situ conservation should be adopted for the natural population of *G. paucinervis* as soon as possible; for example, strictly prohibiting illegal logging, conducting regular inspections of priority protected areas and providing moderate shading during artificial seedling cultivation. We may also establish a seed orchard and germplasm resource bank for *G. paucinervis.*

## Conclusion

The suitable habitat of *G. paucinervis* was located in most regions of Guangxi and Guangdong, the northern region of Hainan, the southern region of Guizhou adjacent to Guangxi, the southern and western regions of Yunna, the southeast region of Sichuan, the northwest region of Hunan, the northeast region of Chongqing, the southwest region of Hubei and the junction area among Hubei, Henan, and Anhui under current climate conditions; (2) four key factors affecting the geographical distribution of this species are the precipitation of the driest quarter (Bio-17) ranging from 33.3 to 133.3 mm; the precipitation of the warmest quarter (Bio-18) from 667.67 to 1000 mm; the annual mean temperature (Bio-01) from 18.0 to 24.0 °C and the annual precipitation (Bio-12) from 1250.0 to 1760.0 mm; (3) The geographical distribution area will gradually decrease with climate warming. In the 2050s, most of Guangxi and Guangdong, southern Guizhou, eastern Yunnan adjacent to Guangxi, southeastern Sichuan, and northern Hainan were identified as stable suitable habitats for *G. paucinervis.* The expanded habitat was located only in the western and southern regions of Yunnan, while the shrunken habitat was at the junction between Guangdong, Fujian, and Jiangxi; Guizhou, Chongqing, and Hunan; Anhui, Henan, and Hubei; the southeastern region of Sichuan; the western region of Hubei; and the adjacent area between Chongqing and Sichuan on their eastern border. By the 2070s the shrinking habitats will have increase in central Guizhou, the northern region of Guangdong and Guangxi, eastern Guangdong, and southern Jiangxi Province. In summary, stable and expanded habitats may be used for the protection, restoration, and development of *G. paucinervis* whereas the unsuitable and shrunken habitats mentioned above should be avoided to reduce the investment risk of blind expansion in the future.

## Supporting information

S1 File Distribution points of *G. paucinervis* in the entire land area of China. (XLSX)
